# Supplementary material for: A Population Dynamic Model to Assess the Diabetes Screening and Reporting Programs and Project the Burden of Undiagnosed Diabetes in Thailand
Source: Int J Environ Res Public Health. 2019 Jun 21;16(12):2207. doi: 10.3390/ijerph16122207 (PMC6617291; doi:10.3390/ijerph16122207)
Supplement: Supplementary file 1 [file ijerph-16-02207-s001.pdf]

Supplementary file

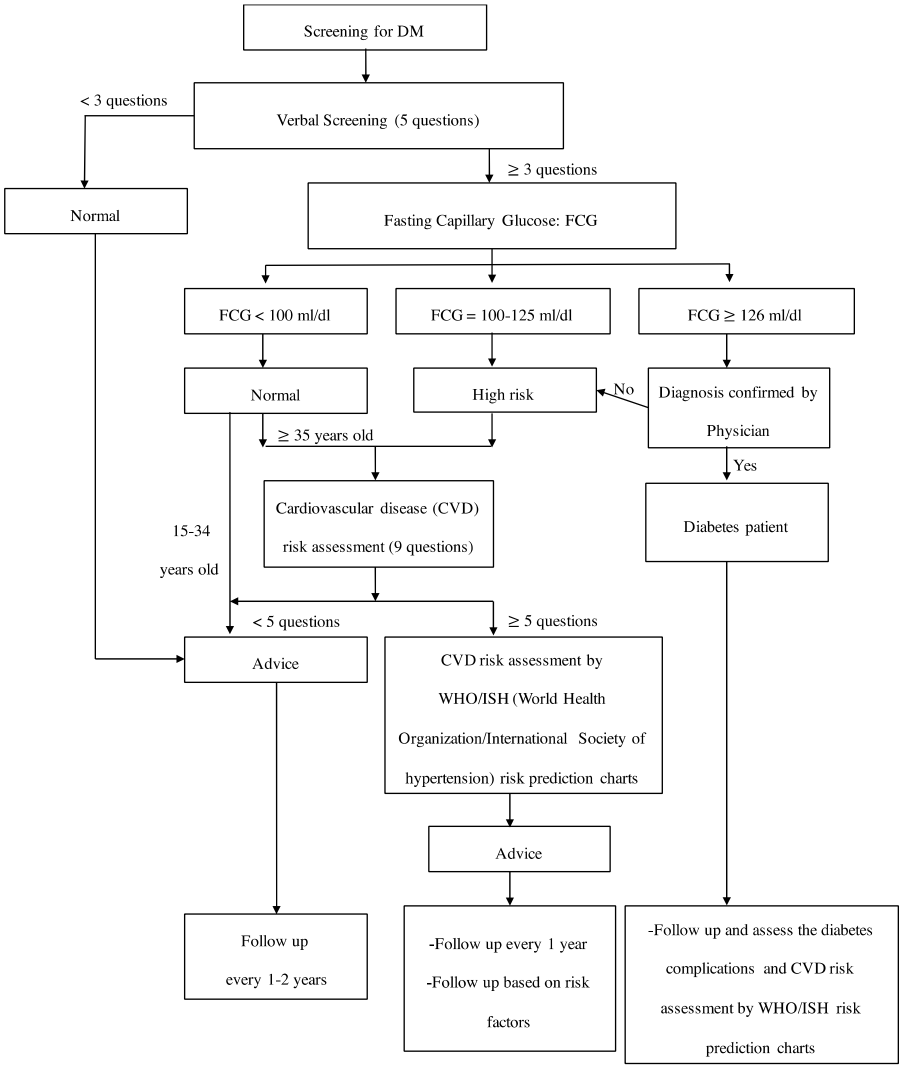

Figure S1. Schematic diagram of screening guidelines for diabetes among the Thai population aged 15 years old or over.

Population aged 15 years old were screened as follow: they were first screened by verbal screening using 5 questions of risk assessment including obesity, hypertension, smoking, family history of diabetes, and neck or armpit crease. If the answer is yes to 3 out of 5 questions, they would be tested by fasting capillary glucose (FCG). FCG is graded into three groups including normal, high risk, and

diabetes confirmed by physician. Those people with normal and high FCG was further assessed by verbal screening using 9 questions of cardiovascular disease risk assessment. If the answer is yes to 5 out of 9 questions, they will be advised and followed up annually [1].

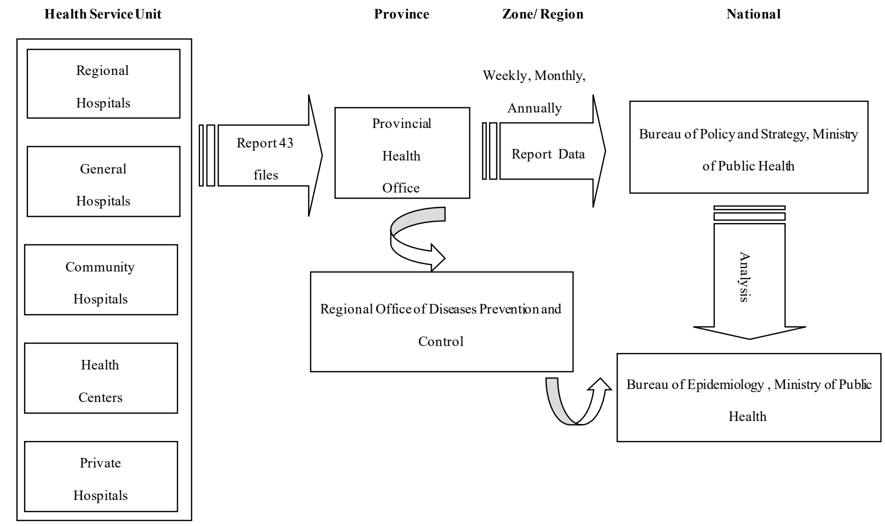

Figure S2. Schematic diagram of Epidemiological Surveillance Report since 2012.

The 12 files system is a data set containing of individual outpatient and inpatient service data from hospitals. The 18 files system is a data set containing of individual outpatient data and health promotion and illness prevention services provided by primary care units and health centers. However, the 18 files data set has been revised to include more health data including of referral data, accident and emergency data. From 2014 to date, 43 data files have been included all data are finally reached and analyzed by Bureau of Epidemiology, Ministry of Public Health.

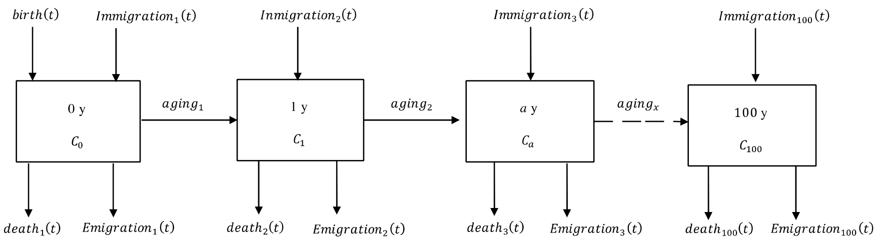

Figure S3. Schematic representation of the demographic deterministic model.

Information S1. Solved a large set of Ordinary Differential Equations (ODE) of Demographic sub-model

We solved a large set of Ordinary Differential Equations (ODE) of demographic deterministic sub-model. Let $C_a(t)$ be the number of people of at age, *a*, at time, *t* and fr_a be the fertility rate in female aged *a* years old [2] The number of newborn babies at any time *t* is shown as follows:

$$birth(t) = \sum_a fr_a . C_a \ (t) \quad \text{(Equation 1)}$$

Death [3] among male and female population were calculated from the age-specific mortality rate dra:

$$death_{ga}(t) = dr_a . C_{ga}(t) \quad \text{(Equation 2)}$$

Net migration [4] among male and female population were calculated from the migration rate mra:

$$migration_{ga}(t) = mr_a . C_{ga}(t) \quad \text{(Equation 3)}$$

Aging is a rate at which individuals move to the next age group were also represented as at rate $\frac{1}{(age.diff)}$ per year where age.diff represented the difference between two age classes. In this model the age.diff is always equal to 1 year. We generated the matrix equation for individual dynamics as follow:

$$\begin{bmatrix} C_1(t+1) \\ C_2(t+1) \\ C_3(t+1) \\ \vdots \\ C_{101}(t+1) \end{bmatrix} = \begin{bmatrix} birth(t) \\ 0 \\ 0 \\ 0 \\ 0 \end{bmatrix} + \begin{bmatrix} migration_{\ 1}(t) \\ migration_{\ 2}(t) \\ migration_{\ 3}(t) \\ \vdots \\ migration_{\ 101}(t) \end{bmatrix} +$$
$$\begin{bmatrix} -aging_1 & 0 & 0 & \cdots & 0 \\ aging_1 & -aging_2 & 0 & \cdots & 0 \\ 0 & aging_2 & -aging_3 & \cdots & 0 \\ \vdots & \vdots & \vdots & \ddots & \vdots \\ 0 & \cdots & \cdots & aging_{100} & -aging_{101} \end{bmatrix} \begin{bmatrix} C_1(t) \\ C_2(t) \\ C_3(t) \\ \vdots \\ C_{101}(t) \end{bmatrix} - \begin{bmatrix} death_{\ 1}(t) \\ death_{\ 2}(t) \\ death_{\ 3}(t) \\ \vdots \\ death_{\ 101}(t) \end{bmatrix}$$

(Equation 4)

All the parameters included in the model was shown in table 1;

Table S1. Parameter table for Diabetes dynamic model.

Parameter	Symbol	Value<br>(95% Credible Interval)	Source/<br>Reference
Population parameters			
Fertility rate by age	fr_a		Census data [2]
Mortality rate by age	dr_a		Census data [3]
Net international migration rate by age	mr_a		Thailand Migration Report [4]
Diabetes parameters			
Case fatality rate of undiagnosed diabetes by age groups (per capita per year)	dmr_{ga}	Aged 0-14 = 0.0009(0.0008-0.001)<br>Aged 15-39 =2.16(2.01-2.25)<br>Aged 40-49 =0.44(0.41-0.46)<br>Aged 50-59 =0.39(0.38-0.43)<br>Aged >= 60 =0.001(0.0005-0.01)	[5]
The diagnosed diabetes rate of each age group among females (per capita per year)	D_{fa}^{DM}	Aged 0-39 = 0.001(0.0009-0.0012)<br>Aged 40-49 =0.011(0.01-0.012)<br>Aged 50-59 =0.032(0.031-0.033)<br>Aged >= 60 =0.026(0.025-0.027)	[6, 7]
The diagnosed diabetes rate of each age group among males (per capita per year)	D_{ma}^{DM}	Aged 0-39 = 0.005(0.004-0.006)<br>Aged 40-49 =0.017(0.016-0.018)<br>Aged 50-59 =0.021(0.02-0.022)<br>Aged >= 60 =0.018(0.017-0.019)	[6, 7]
Percentage of Reporting diabetes	Report	Report (in 2005-2009) = 84.2% (84.1-84.3%)<br>Report (in 2010-2014) = 85.7% (85.5-85.8%)<br>Report (in 2015) =<br>87.4% (87.2-87.5%)	Estimated

Parameter	Symbol	Value<br>(95% Credible Interval)	Source/<br>Reference
The diabetes positive screening rate of each age group among females (per capita per year)	S_{fa}^{DM}	In 2005-2009<br>Aged 0-14 = 0 (fixed)<br>Aged 15-34 = 9.49(8.19-10.33)<br>Aged 35-49 =1.33(1.27-1.39)<br>Aged 50-59=0.97(0.92-1.04)<br>Aged >= 60 =0.83(0.79-0.85)<br><br>In 2010-2015<br>Aged 0-14 = 0 (fixed)<br>Aged 15-34 = 3.70(3.39-3.80)<br>Aged 35-49 =1.32(1.21-1.38)<br>Aged 50-59=0.82(0.77-0.88)<br>Aged >= 60 =0.81(0.78-0.84)	Estimated
The diabetes positive screening rate of each age group among males (per capita per year)	S_{ma}^{DM}	In 2005-2009<br>Aged 0-14 = 0 (fixed)<br>Aged 15-34 =0.06(0.05-0.07)<br>Aged 35-49 =0.25(0.24-0.26)<br>Aged 50-59=0.49(0.47-0.53)<br>Aged >= 60 =1.07(1.02-1.14)<br><br>In 2010-2015<br>Aged 0-14 = 0 (fixed)<br>Aged 15-34 = 0.06(0.05-0.07)<br>Aged 35-49 =0.28(0.26-0.29)<br>Aged 50-59=0.64(0.61-0.69)<br>Aged >= 60 =1.11(1.06-1.19)	Estimated
Case fatality rate of undiagnosed diabetes (per capita per year)	$dumr_a$	Aged 0-14 = 0.0009(0.0008-0.001)<br>Aged 15-39 =2.16(2.01-2.25)<br>Aged 40-49 =0.44(0.41-0.46)<br>Aged 50-59 =0.39(0.38-0.43)<br>Aged >= 60 =0.001(0.0005-0.005)	Estimated

Parameter	Symbol	Value<br>(95% Credible Interval)	Source/<br>Reference
The diabetes incidence rate of each age group among females (per capita per year)	K_{fa}^{DM}	Aged 0-39 = 0.001(0.0009-0.0012)<br>Aged 40-49 =0.011(0.01-0.012)<br>Aged 50-59 =0.032(0.031-0.033)<br>Aged >= 60 =0.026(0.025-0.027)	Estimated
The diabetes incidence rate of each age group among males (per capita per year)	K_{ma}^{DM}	Aged 0-39 = 0.005(0.004-0.006)<br>Aged 40-49 =0.017(0.016-0.018)<br>Aged 50-59 =0.021(0.02-0.022)<br>Aged >= 60 =0.018(0.017-0.019)	Estimated

Information S2. Solved a large set of Ordinary Differential Equations (ODE) of Diabetes dynamic sub-model.

Case fatality ($deathDM\_un_{qa}$) of undiagnosed, diabetic individuals in each age group were a sum of the deaths from natural causes (dr_a) and the deaths occurred from DM itself with the case fatality rates ($dumr_a$):

$$deathDM\_un_{ga}(t) = (dr_a + dumr_a).C_{ga}^{DM\_un}(t) \quad \text{(Equation 5)}$$

Since the case fatality data were not stratified by gender, it was assumed that the rates were the same in both genders.

Case fatality of diabetic ($deathDM_{ga}$) [5] among diagnosed individuals in each age group were a sum of the deaths from natural causes (dr_a) and the deaths occurred from DM itself with the case fatality rates dmr_a:

$$deathDM_{ga}(t) = (dr_a + dmr_a).C_{ga}^{DM}(t) \quad \text{(Equation 6)}$$

The diabetes incidence of each gender and age group (inc_{ga}) was taken to be a function of nondiabetic (C_{ga}^{H}) with corresponding diabetes incidence rate K_{ga}^{DM} which represents the rates of total diabetes both diagnosed and undiagnosed of each age group:

$$inc_{ga}(t) = K_{ga}^{DM}.C_{ga}^{H}(t) \quad \text{(Equation 7)}$$

The positive diabetes screening of each age group ($Screening_{ga}$) was taken to be a function of undiagnosed diabetes ($C_{ga}^{DM\_un}$) with corresponding diabetes screening rate S_{ga}^{DM} of each gender and age group:

$$Screening_{ga}(t) = S_{ga}^{DM} . C_{ga}^{DM\_un}(t) \quad \text{(Equation 8)}$$

The positive diagnosed diabetes of each age group ($Diagnosis_{ga}$) was taken to be a function of undiagnosed diabetes ($C_{ga}^{DM\_un}$) with corresponding diagnosed diabetes rate D_{ga}^{DM} of gender and each age group:

$$Diagnosis_{ga}(t) = D_{ga}^{DM} . C_{ga}^{DM\_un}(t) \quad \text{(Equation 9)}$$

Rates of change in C_{ga}^{H}, $C_{ga}^{DM\_un}$ and C_{ga}^{DM} within the gender g and age group a were represented by ordinary differential equations. For example, the rate of change of the nondiabetic females aged 1 year old was represented by the following equation which describes the balance between birth inflows, diabetes incidence, aging, and death as follows:

$$\frac{dC_{f1}^{H}}{dt} = birth_f - inc_{f1} - death_{f1} - aging_1 C_{f1}^{H} + migration_{f1} \quad \text{(Equation 10)}$$

Similarly, rate of change in the undiagnosed diabetic compartment was calculated as a balance between screening, diagnosis, diabetes incidence and death outflows as follows:

$$\frac{dC_{f1}^{DM\_un}}{dt} = inc_{f1} - death_{f1} - deathDM\_un_{f1} - Screening_{f1} - Diagnosis_{f1} - aging_1 C_{f1}^{DM_{un}} + migration_{f1}$$

(Equation 11)

Rate of change in the diabetic compartment was calculated as a balance between screening rate, diagnosis, aging, and death outflows as follow:

$$\frac{dC_{f1}^{DM}}{dt} = Screening_{f1} + Diagnosis_{f1} - death_{f1} - deathDM_{f1} - aging_1 C_{f1}^{DM} + migration_{f1}$$

(Equation 12)

C_{ga}^{H} , $C_{ga}^{DM\_un}$ and C_{ga}^{DM} were determined by numerical integration of the corresponding differential equations. Diabetes prevalence in any gender g and age group a (PRV_{ga}) was finally calculated as following:

$$PRV_{ga} = \frac{C_{ga}^{DM\_un} + C_{ga}^{DM}}{C_{ga}^{H} + C_{ga}^{DM\_un} + C_{ga}^{DM}} \quad \text{(Equation 13)}$$

Cumulative incidence was analyzed by numerical integration of the corresponding healthy (C_{ga}^{H}) and diabetes incidence of each age group (K_{ga}^{DM}) calculated as following:

$$CumInc_{ga} = \int_{t}^{t+1} C_{ga}^{H} \times K_{ga}^{DM} dt \quad \text{(Equation 14)}$$

and similarly, for other reported measures. Note that the report parameters were used to calculate reported incidence and prevalence diabetes by multiplication of diagnosed incidence and prevalence diabetes and reporting proportion.

Information S3. The Bayesian framework.

Bayesian inference of diabetes dynamic model provides a framework for estimating parametric uncertainty in terms of probabilistic distributions, and allowing a direct quantification of parameter uncertainty.

Bayes theorem states that the best estimate (posterior uncertainty $p(\theta|y)$) for a parameter vector θ given data y is given by:

$$p(\theta|y) = \frac{p(\theta)p(y|\theta)}{p(y)} \qquad \text{(Equation 15)}$$

Here, $p(\theta)$ is the prior information and, $\frac{p(y|\theta)}{p(y)}$ is the likelihood ratio. Markov Chain Monte Carlo (MCMC) algorithms were applied to approximate these distributions which used a sampling scheme to estimate the posterior distribution [8, 9].

Prior distribution

Uniform distribution was chosen to be the prior distribution for all parameter values given little information about these parameters was measured and reported. The minimum and maximum values were initiated and narrowed down from the iterative model fitting procedure.

Likelihood function

The likelihood of parameters given the data is equal to probability of the data given the parameters including incidence rates, screening and reporting. We defined the likelihood as the product of likelihood terms for each data point. The data arise from the diabetes annual epidemiological surveillance report between 2005 and 2015, and are linked to the summation of expected age and gender rates via a Poisson distribution. The log-likelihood (used as the target in the MCMC algorithm) is:

$$LL = \sum_a \left(\sum_t \log \left(\frac{DM^{\theta} \exp(-\theta)}{\theta!} \right) \right) \qquad \text{(Equation 16)}$$

Where θ is the annual diabetes data at each age class a and time t and DM is the expected incidences from the model at each age class a and time t.

Posterior estimation

We used Differential-Evolution MCMCzs (DE-MCzs) to estimate the posterior distributions. We consider Markov chain methods of sampling that are proposed by Ter Braak and Vrugt et al, 2008 [10], which has been used for numerical problems, implemented in the Bayesian Tools R package. Differential Evolution Markov Chain (DE-MC) is an adaptive MCMC algorithm, in which multiple chains are run in parallel and presented. The DE-MCzs combines characteristics of conventional MCMC methods with the ideas of differential evolution optimization algorithms by making use of the full joint density function and (independent) proposal distributions for each of the variables including reporting, screening and incidence rate of diabetes. These samples are accepted probabilistically based on the acceptance probability. Uniform distributions centered at the current state of the chain. This proposal distribution randomly perturbs the current state of the chain, and then either accepts or rejects the perturbed value. Six separate chains, each consisting of 35,000 iterations, were run in parallel, are shown in Supplementary Figure 6.

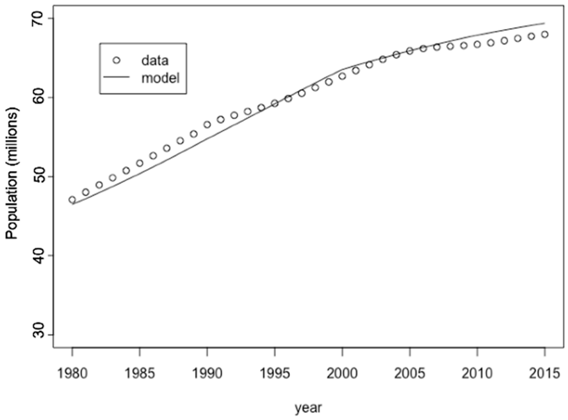

Figure S4. Projection of the population size of Thailand between 1980 and 2015. White dot, total population each year; line, model.

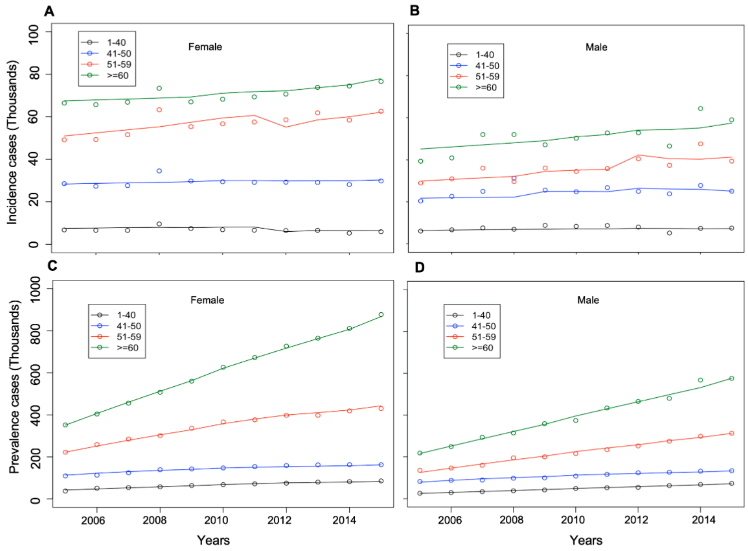

Figure S5. Annual epidemiological surveillance report of diabetes incidence and prevalence in Thailand between 2005 and 2015 by age; (A) incidence among female (top left) and (B) male (top right), (C) prevalence among female (bottom left) and (D) male (bottom right), respectively and the fitted diabetes model. Line and dots represent model and data.

Deleted:

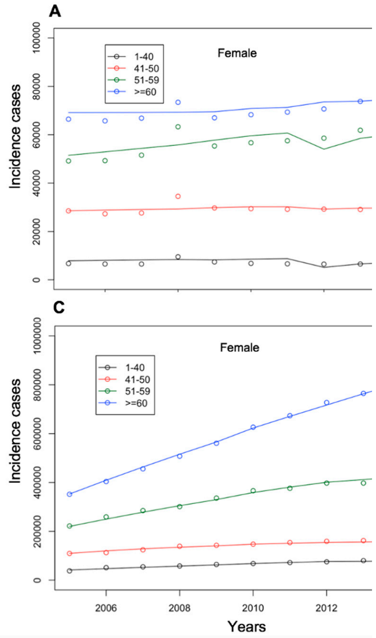

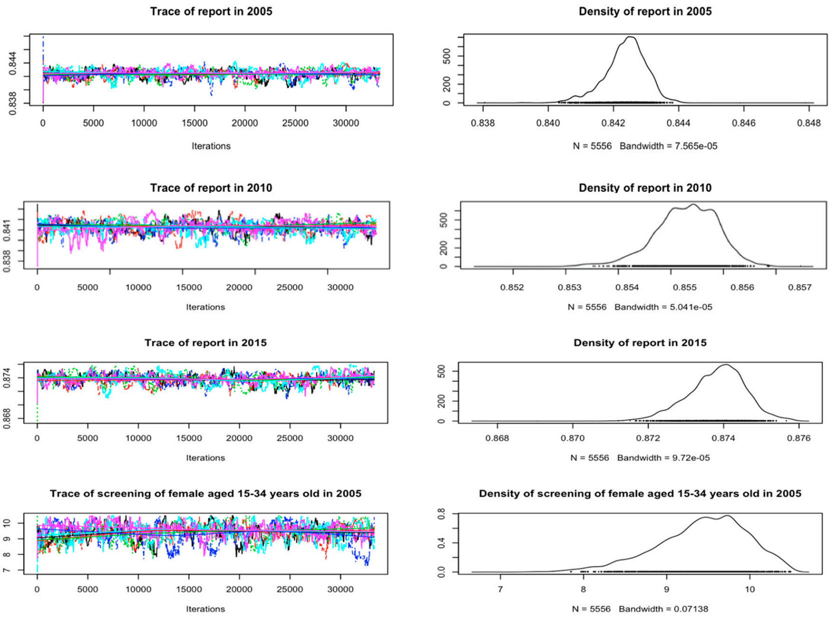
Trace of report in 2005
Iterations
Density of report in 2005
N = 5556 Bandwidth = 7.565e-05
Trace of report in 2010
Iterations
Density of report in 2010
N = 5556 Bandwidth = 5.041e-05
Trace of report in 2015
Iterations
Density of report in 2015
N = 5556 Bandwidth = 9.72e-05
Trace of screening of female aged 15-34 years old in 2005
Iterations
Density of screening of female aged 15-34 years old in 2005
N = 5556 Bandwidth = 0.07138

Trace of screening of female aged 35-49 years old in 2005

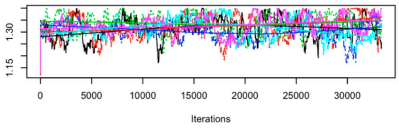

Density of screening of female aged 35-49 years old in 2005

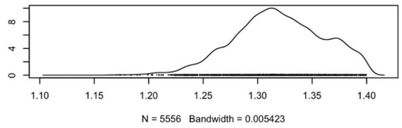

Trace of screening of female aged 50-59 years old in 2005

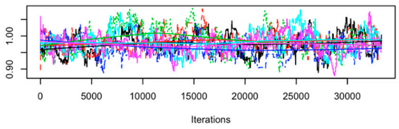

Density of screening of female aged 50-59 years old in 2005

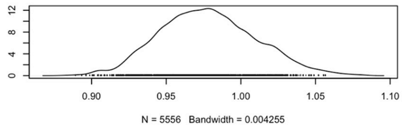

Trace of screening of female aged over 59 years old in 2005

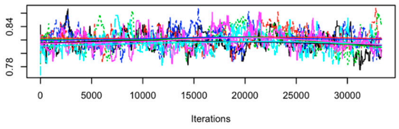

Density of screening of female aged over 59 years old in 2005

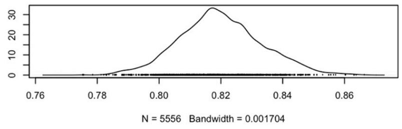

Trace of screening of female aged 15-34 years old in 2015

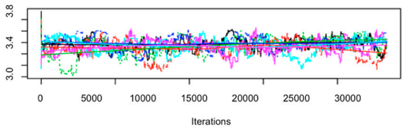

Density of screening of female aged 15-34 years old in 2015

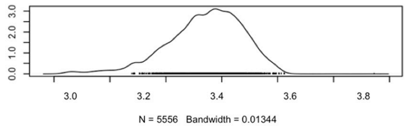

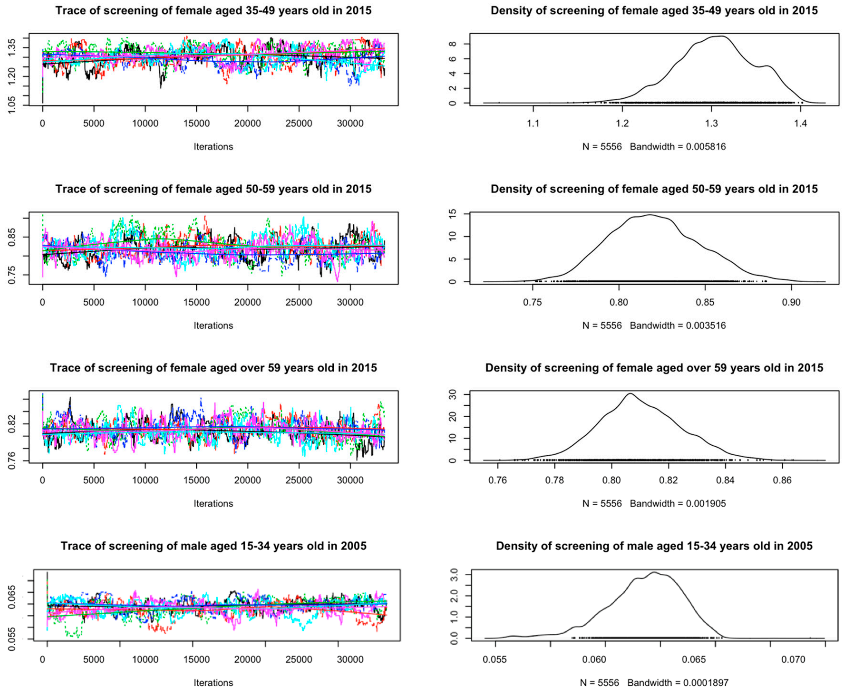
Trace of screening of female aged 35-49 years old in 2015
Iterations
Density of screening of female aged 35-49 years old in 2015
N = 5556 Bandwidth = 0.005816
Trace of screening of female aged 50-59 years old in 2015
Iterations
Density of screening of female aged 50-59 years old in 2015
N = 5556 Bandwidth = 0.003516
Trace of screening of female aged over 59 years old in 2015
Iterations
Density of screening of female aged over 59 years old in 2015
N = 5556 Bandwidth = 0.001905
Trace of screening of male aged 15-34 years old in 2005
Iterations
Density of screening of male aged 15-34 years old in 2005
N = 5556 Bandwidth = 0.0001897

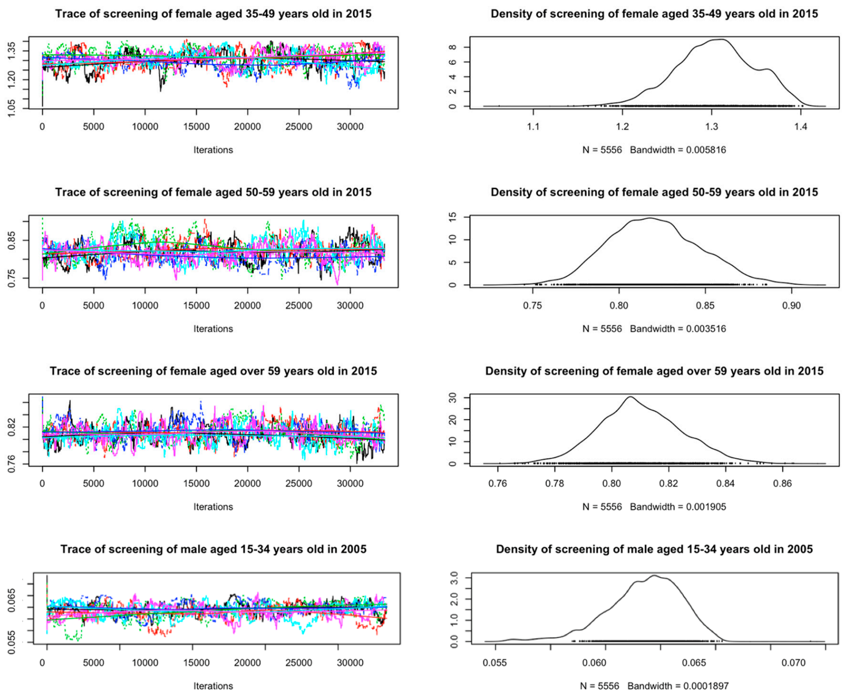
Trace of screening of female aged 35-49 years old in 2015
Iterations
Density of screening of female aged 35-49 years old in 2015
N = 5556 Bandwidth = 0.005816
Trace of screening of female aged 50-59 years old in 2015
Iterations
Density of screening of female aged 50-59 years old in 2015
N = 5556 Bandwidth = 0.003516
Trace of screening of female aged over 59 years old in 2015
Iterations
Density of screening of female aged over 59 years old in 2015
N = 5556 Bandwidth = 0.001905
Trace of screening of male aged 15-34 years old in 2005
Iterations
Density of screening of male aged 15-34 years old in 2005
N = 5556 Bandwidth = 0.0001897

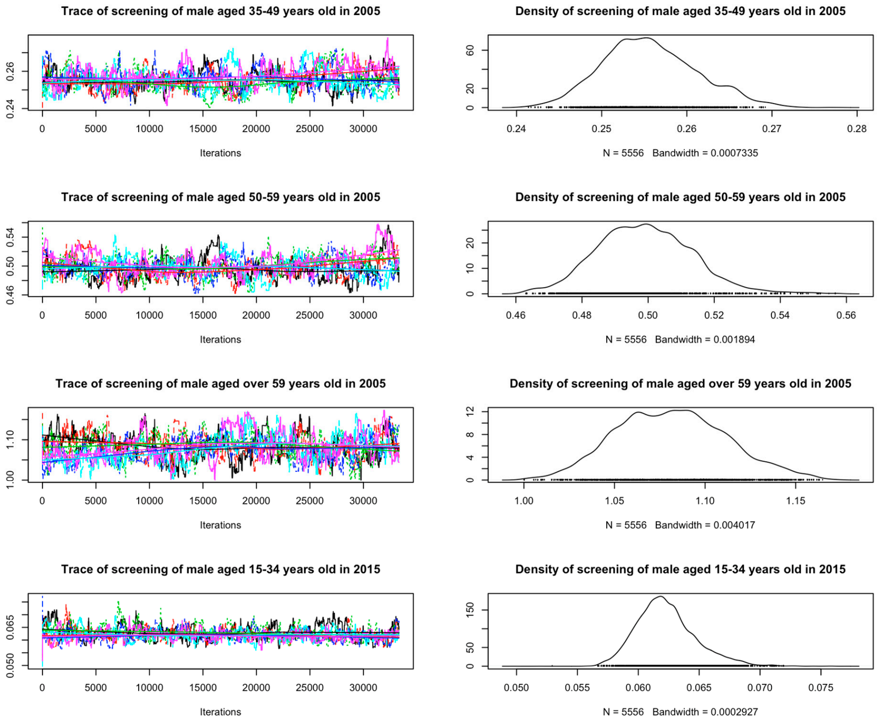
Trace of screening of male aged 35-49 years old in 2005
Iterations
Density of screening of male aged 35-49 years old in 2005
N = 5556 Bandwidth = 0.0007335
Trace of screening of male aged 50-59 years old in 2005
Iterations
Density of screening of male aged 50-59 years old in 2005
N = 5556 Bandwidth = 0.001894
Trace of screening of male aged over 59 years old in 2005
Iterations
Density of screening of male aged over 59 years old in 2005
N = 5556 Bandwidth = 0.004017
Trace of screening of male aged 15-34 years old in 2015
Iterations
Density of screening of male aged 15-34 years old in 2015
N = 5556 Bandwidth = 0.0002927

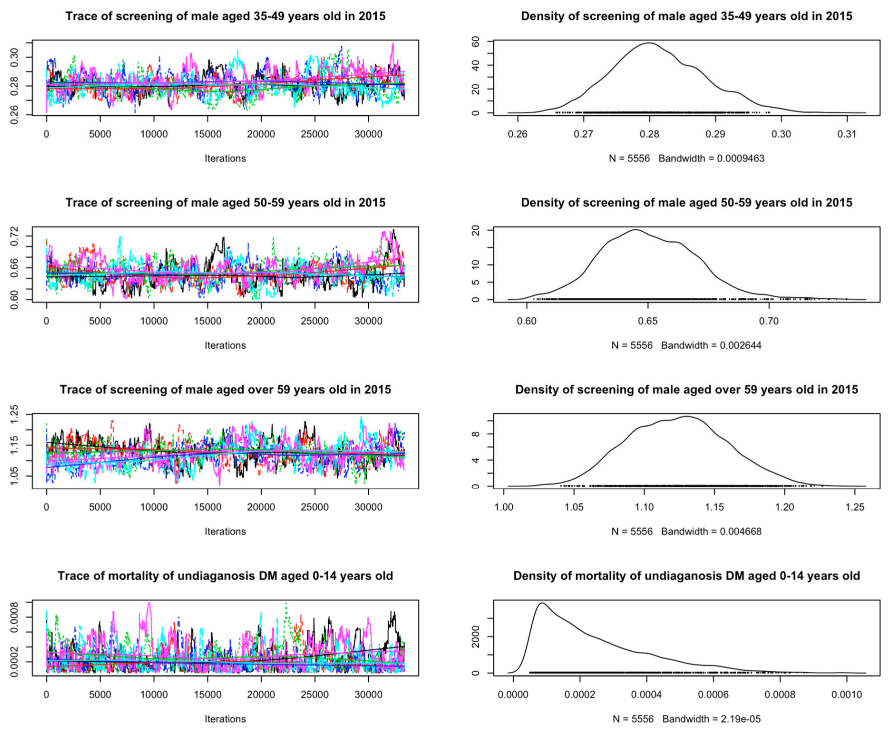
Trace of screening of male aged 35-49 years old in 2015
Iterations
Density of screening of male aged 35-49 years old in 2015
N = 5556 Bandwidth = 0.0009463
Trace of screening of male aged 50-59 years old in 2015
Iterations
Density of screening of male aged 50-59 years old in 2015
N = 5556 Bandwidth = 0.002644
Trace of screening of male aged over 59 years old in 2015
Iterations
Density of screening of male aged over 59 years old in 2015
N = 5556 Bandwidth = 0.004668
Trace of mortality of undiaganosis DM aged 0-14 years old
Iterations
Density of mortality of undiaganosis DM aged 0-14 years old
N = 5556 Bandwidth = 2.19e-05

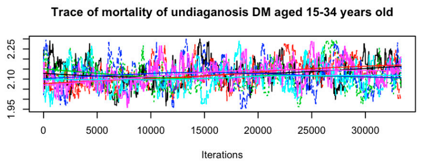
Trace of mortality of undiaganosis DM aged 15-34 years old
Iterations

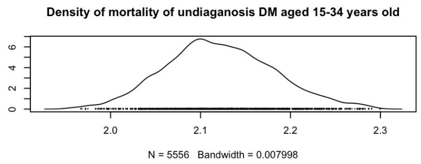
Density of mortality of undiaganosis DM aged 15-34 years old
N = 5556 Bandwidth = 0.007998

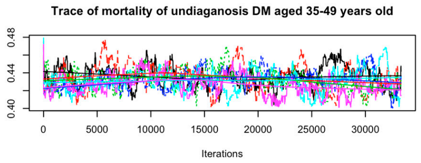
Trace of mortality of undiaganosis DM aged 35-49 years old
Iterations

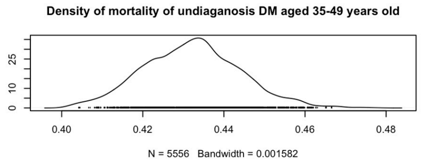
Density of mortality of undiaganosis DM aged 35-49 years old
N = 5556 Bandwidth = 0.001582

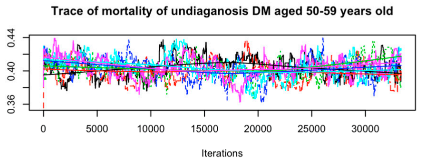
Trace of mortality of undiaganosis DM aged 50-59 years old
Iterations

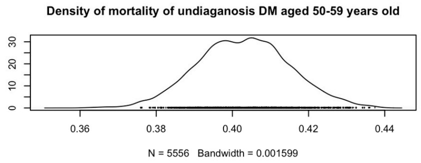
Density of mortality of undiaganosis DM aged 50-59 years old
N = 5556 Bandwidth = 0.001599

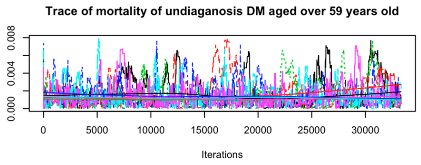
Trace of mortality of undiaganosis DM aged over 59 years old
Iterations

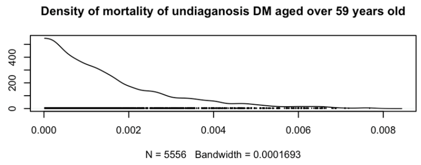
Density of mortality of undiaganosis DM aged over 59 years old
N = 5556 Bandwidth = 0.0001693

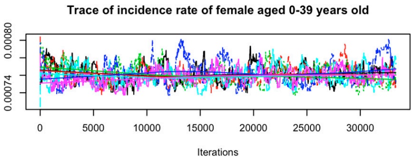
Trace of incidence rate of female aged 0-39 years old
0.00074
0.00080
0 5000 10000 15000 20000 25000 30000
Iterations

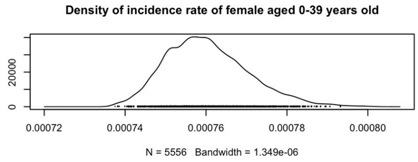
Density of incidence rate of female aged 0-39 years old
0
20000
0.00072 0.00074 0.00076 0.00078 0.00080
N = 5556 Bandwidth = 1.349e-06

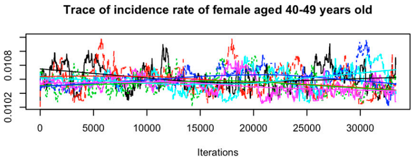
Trace of incidence rate of female aged 40-49 years old
0.0102
0.0108
0 5000 10000 15000 20000 25000 30000
Iterations

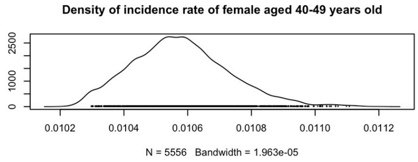
Density of incidence rate of female aged 40-49 years old
0
1000
2500
0.0102 0.0104 0.0106 0.0108 0.0110 0.0112
N = 5556 Bandwidth = 1.963e-05

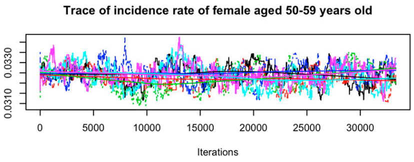
Trace of incidence rate of female aged 50-59 years old
0.0310
0.0320
0.0330
0 5000 10000 15000 20000 25000 30000
Iterations

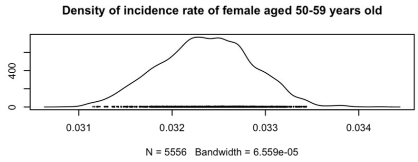
Density of incidence rate of female aged 50-59 years old
0
400
0.031 0.032 0.033 0.034
N = 5556 Bandwidth = 6.559e-05

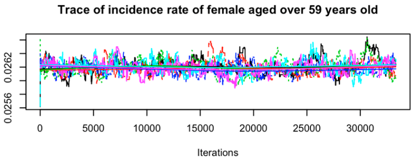
Trace of incidence rate of female aged over 59 years old
0.0256
0.0262
0 5000 10000 15000 20000 25000 30000
Iterations

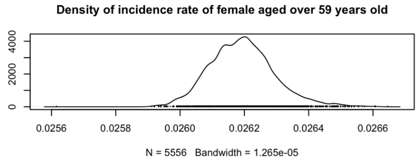
Density of incidence rate of female aged over 59 years old
0
2000
4000
0.0256 0.0258 0.0260 0.0262 0.0264 0.0266
N = 5556 Bandwidth = 1.265e-05

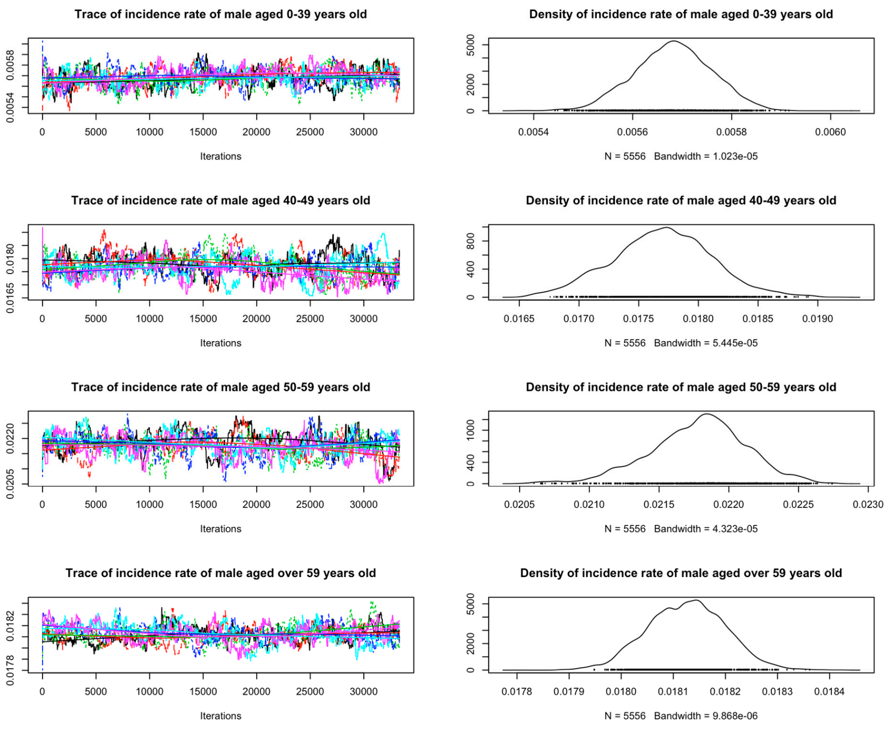

Figure S6. Posterior distributions from the diabetes model, that each row corresponds to the separate parameter, the left-hand column contains traces with 6 color chains (dashed lines: actual traces, solid lines: trends) and the right-hand column contains the posterior distribution, corresponding to each parameter.

Table S2. Proportion of reporting estimates (95% credible interval) each 5-year interval.

**Parameters**	**Years**		
	**2005-2009**	**2010-2014**	**2015-now**
**Proportion of Reporting**	**0.843 ± 0.002**	**0.857 ± 0.002**	**0.874 ± 0.002**

Table S3. Among both males and females, the estimates and 95% credible interval of screening rates and fix proportion of reporting each gender.

Year	Age group							
	15-34		35-49		50-59		≥60	
	Male	Female	Male	Female	Male	Female	Male	Female
2005-2012	0.06 ± 0.01	9.43 ± 0.51	0.28 ± 0.01	1.33 ± 0.06	0.65 ± 0.04	0.97 ± 0.04	1.07 ± 0.07	0.82 ± 0.03
2013-now	0.06 ± 0.01	3.73 ± 0.09	0.28 ± 0.01	1.32± 0.06	0.65 ± 0.03	0.82 ± 0.05	1.11 ± 0.08	0.82 ± 0.02

Table S4. Case fatality rate of undiagnosed diabetes estimates (95% credible interval) a 10-year interval by age classes.

Parameters	Age classes				
	1-14	15-39	40-49	50-59	≥60
Case fatality rate of undiagnosed diabetes	0.001±0.0005	2.16 ± 0.08	0.44± 0.02	0.39 ± 0.03	0.001± 0.004

Table S5. Among both males and females, the estimates and 95% credible interval of diabetes incidence rates a 10-year interval each gender.

Parameters	Age group							
	0-39		40-49		50-59		≥60	
	Male	Female	Male	Female	Male	Female	Male	Female
DM incidence rates	0.006 ± 0.0004	0.001 ± 0.0004	0.017 ± 0.001	0.011 ± 0.001	0.021 ± 0.001	0.033 ± 0.001	0.018 ± 0.001	0.026 ± 0.0003

Table S6. Estimations (in thousands) of the number of males and females with undiagnosed diabetes by age group for selected years, using increasing incidence rates combined with The Population and Housing Census of Thailand.

year	Age-group (years)								Total
	0-39		40-49		50-59		≥60		
	Male	Female	Male	Female	Male	Female	Male	Female	
2005	213,000	25,000	87,000	24,000	58,000	56,000	41,000	86,000	590,000
2010	258,000	31,000	87,000	25,000	62,000	63,000	45,000	88,000	659,000
2015	249,000	32,000	83,000	25,000	57,000	77,000	47,000	94,000	664,000

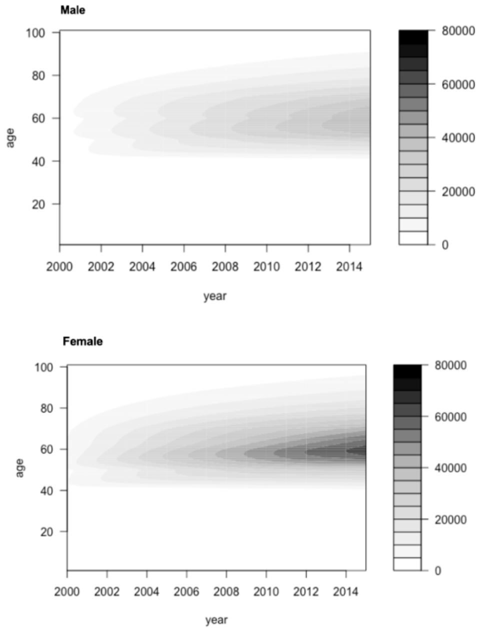

Figure S7. Results of an age-specific diabetes cases dynamic model among both (A) male (top plot) and (B) female (bottom plot) between 2005 and 2015. In both plots, prevalence cases are indicted by the black color with legend at right.

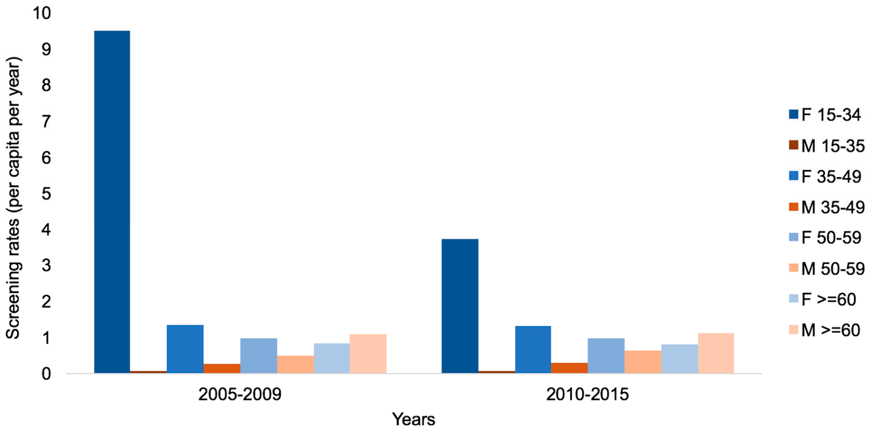

Figure S8. Positive screening rates (per capita per year) among females (blue colors) and male (orange colors) by gender within 5 years intervals between 2005-2009 and 2010-2015.

References

1. WHO *Screening guidelines for diabetes*; Bureau of Non Communicable Diseases: Ministry of Public Health, Thailand, 2013.
2. MoPH, *Public health statistics A.D.2010*. Ministry of Public health: Thailand, 2010.
3. MoPH, *Public health statistics A.D.2000*. Ministry of Public health: Thailand, 2000.
4. Huguet, J. W. *Thailand Migration Report 2011*; International Organization for Migration: Thailand, 2011.
5. Pratipanawatr, T.; Rawdaree, P.; Chetthakul, T.; Bunnag, P.; Ngarmukos, C.; Benjasuratwong, Y.; Leelawatana, R.; Kosachunhanun, N.; Plengvidhya, N.; Deerochanawong, C.; Suwanwalaikorn, S.; Krittiyawong, S.; Mongkolsomlit, S.; Komoltri, C., Thailand Diabetic Registry cohort: predicting death in Thai diabetic patients and causes of death. *J Med Assoc Thai* **2010,** 93 Suppl 3, S12-20.
6. Thonghong, A. *Annual Epidemilological Surveillance Report*; Bureau of Epidemiology: Ministry of Public Health, Thailand, 2015.
7. Papier, K.; Jordan, S.; D'Este, C.; Bain, C.; Peungson, J.; Banwell, C.; Yiengprugsawan, V.; Seubsman, S. A.; Sleigh, A., Incidence and risk factors for type 2 diabetes mellitus in transitional Thailand: results from the Thai cohort study. *BMJ Open* **2016,** 6, (12), e014102.
8. Augustynczik, A. L. D.; Hartig, F.; Minunno, F.; Kahle, H.-P.; Diaconu, D.; Hanewinkel, M.; Yousefpour, R., Productivity of Fagus sylvatica under climate change – A Bayesian analysis of risk and uncertainty using the model 3-PG. *Forest Ecology and Management* **2017,** 401, (Supplement C), 192-206.
9. Hartig, F., Minunno, F., Paul, S., BayesianTools: General-Purpose MCMC and SMC Samplers and Tools for Bayesian Statistics. R package version. R package version 0.1.3. https://cran.r-project.org/web/packages/BayesianTools/ndex.html

10. Ter Braak, C. J., Vrugt, J.A., Differential evolution Markov chain with snooker updater and fewer chains. *Stat. Comput.* **2008,** 18, 435–446.
